# Neonatal mortality prediction with routinely collected data: a machine learning approach

**DOI:** 10.1186/s12887-021-02788-9

**Published:** 2021-07-21

**Authors:** André F. M. Batista, Carmen S. G. Diniz, Eliana A. Bonilha, Ichiro Kawachi, Alexandre D. P. Chiavegatto Filho

**Affiliations:** 1 Department of Epidemiology, School of Public Health, University of São Paulo, 715 Av Dr Arnaldo, Sao Paulo, SP 01246-904 Brazil; 2 Department of Health, Life Cycles and Society, School of Public Health, University of São Paulo, Sao Paulo, Brazil; 3 Municipal Department of Health of São Paulo, Sao Paulo, Brazil; 4 Department of Social and Behavioral Sciences, Harvard T.H. Chan School of Public Health, Harvard University, Cambridge, USA

**Keywords:** Machine learning, Artificial intelligence, Prediction, Neonatal mortality, Birth records, Brazil

## Abstract

**Background:**

Recent decreases in neonatal mortality have been slower than expected for most countries. This study aims to predict the risk of neonatal mortality using only data routinely available from birth records in the largest city of the Americas.

**Methods:**

A probabilistic linkage of every birth record occurring in the municipality of São Paulo, Brazil, between 2012 e 2017 was performed with the death records from 2012 to 2018 (1,202,843 births and 447,687 deaths), and a total of 7282 neonatal deaths were identified (a neonatal mortality rate of 6.46 per 1000 live births). Births from 2012 and 2016 (*N* = 941,308; or 83.44% of the total) were used to train five different machine learning algorithms, while births occurring in 2017 (*N* = 186,854; or 16.56% of the total) were used to test their predictive performance on new unseen data.

**Results:**

The best performance was obtained by the extreme gradient boosting trees (XGBoost) algorithm, with a very high AUC of 0.97 and F1-score of 0.55. The 5% births with the highest predicted risk of neonatal death included more than 90% of the actual neonatal deaths. On the other hand, there were no deaths among the 5% births with the lowest predicted risk. There were no significant differences in predictive performance for vulnerable subgroups. The use of a smaller number of variables (WHO’s five minimum perinatal indicators) decreased overall performance but the results still remained high (AUC of 0.91). With the addition of only three more variables, we achieved the same predictive performance (AUC of 0.97) as using all the 23 variables originally available from the Brazilian birth records.

**Conclusion:**

Machine learning algorithms were able to identify with very high predictive performance the neonatal mortality risk of newborns using only routinely collected data.

**Supplementary Information:**

The online version contains supplementary material available at 10.1186/s12887-021-02788-9.

## Background

High rates of infant mortality are a persistent challenge for most developing countries. Despite recent improvements, the Millenium Development Goal (MDG) target of reducing child mortality globally by two thirds between 1990 and 2015 was not achieved [1]. The first 28 days of life, i.e. the neonatal period, is considered the most crucial for child and young adolescent survival. Between 2018 and 2030, there will be an estimated 27.8 million worldwide deaths in the first month of life in case every country maintains their current rate of reduction [2].

Progress in neonatal mortality has been slower than for other young age groups. The global neonatal mortality rate fell 42% from 2000 to 2018 (from 31 to 18 deaths per 1000 live births), while for older children and young adolescent the decrease was of 53% (from 15 to 7 deaths per 1000 children) [3]. In Brazil, neonatal mortality in 2017 was 8.5 deaths per 1000 live births, which is higher than the rate among other upper-middle income countries (7.1 per 1000 live births) [2].

Machine learning models have provided accurate predictions in a variety of settings such as infant growth [4], differentiation of sepsis and non-infectious systemic inflammatory response syndrome (SIRS) in critically ill children [5], and mortality risk in critically-ill patients with cancer [5, 6]. Mortality risk prediction can be especially impactful in the case of neonatal mortality [7], as most cases can be prevented with basic adequate care in low and middle-income countries [8]. However, in order to be readily available for health professionals these predictive algorithms must use as input data collected within the daily routine of healthcare services.

The objective of the study was to use official data from Brazilian birth records to train machine learning models to predict neonatal mortality risk. We also tested the predictive performance of these algorithms using only the minimum set of perinatal indicators from the World Health Organization and then suggested additions to this list.

## Methods

Initially, all live births occurring in the Municipality of São Paulo between 2012 and 2017 (*N* = 1,202,843) were included. Births with missing results and with a gestational age of less than 15 weeks or greater than 45 weeks were excluded, leading to a final sample of 1,128,162 live births (93.79% of the original population). A probabilistic linkage of these live births records with neonatal deaths occurring between 2012 and 2018 was performed through a collaboration with the municipal secretary of health, by using the mother’s name, date of birth and the name of the deceased, and a total of 7282 deaths were identified (a neonatal mortality rate of 6.46 per 1000 live births). The study was approved by the ethics committee of the School of Public Health of the University of São Paulo (CAAE: 98163018.2.0000.5421).

Completion of a birth record is mandatory for every live birth occurring in Brazil. In the case of São Paulo, the municipal Secretary of Health has sought to guarantee its full coverage, which is around 99.8% of total live births [9]. For this study, every birth occurring between 2012 and 2016 (*N* = 941.308; or 83.44% of the total) was used to train the machine learning algorithms, and births occurring in 2017 (*N* = 186.854; or 16.56% of the total) were used to test the predictive performance of these algorithms on new unseen data (test set). The performance of five popular machine learning algorithms (logistic regression, neural networks, extreme gradient boosting trees, lightGBM and catboost) was analyzed on the test set.

With the exception of logistic regression that does not have hyperparameters, all algorithms had their hyperparameters tuned with 10-fold cross-validation with Bayesian optimization (Additional file 1). Predictive performance was assessed using the area under the ROC curve (AUC). Other performance metrics calculated for each algorithm include F1-score, precision (also known as positive predictive value, PPV), negative predictive value, area under the precision recall curve (AUPRC), sensitivity (recall), specificity, and percentage of total deaths included among the 5% highest predicted risk and lowest 5% predicted risk.

We used as predictors all variables available from the Brazilian live birth record: place of delivery (hospital, other health facility, residence, others), health facility type (public or private), age of the mother (in years), sex, 1st minute Apgar score, 5th minute Apgar score, birth weight (in grams), gestational age (in weeks), type of pregnancy (single, double or triple or more), type of delivery (vaginal or cesarean), maternal education, presence of congenital anomaly (yes/no), maternal ethnicity, antenatal visits, month of first antenatal visit, type of presentation (cephalic, breech, transversal or other), induced labor (yes/no), professional that assisted the labor (physician, nurse, midwife or others), number of previous live births, number of previous fetal losses and abortions, number of previous pregnancies, number of previous vaginal deliveries and number of previous cesarean deliveries.

The importance of ensuring predictive fairness for vulnerable population groups has been a growing concern in the application of machine learning algorithms [10, 11]. The algorithm with the best performance (extreme gradient boosting trees), was then applied separately for vulnerable subgroups (non-white mothers, mothers with low education, i.e. less than basic education, and teenage mothers) in order to compare each group’s predictive performance with its complementary group.

The performance of the extreme gradient boosting trees algorithm to identify neonatal mortality risk was also tested by using as predictors only the minimum set of perinatal indicators to be collect for all births, as suggested by the World Health Organization: maternal age, place of delivery, mode of delivery, birth weight and gestational age (in weeks) [12]. In addition, with the aim of suggesting inclusions to this list, we analyzed differences in performance by sequentially adding the three individual variables that contributed the most to improve the predictive performance of the model.

Finally, we also analyzed the predictive performance of deaths of children under 1 year of age (i.e. infant mortality, which also includes the neonatal period, *N* = 10,902), again using births from 2012 to 2016 for training and births from 2017 for testing.

## Results

Table 1 presents the predictive performance on the new unseen data (from 2017) for the five machine learning algorithms (logistic regression, artificial neural networks, extreme gradient boosting trees, lightGBM and catboost). For every predictive measure, the best performance was obtained by the extreme gradient boosting trees (XGBoost) algorithm, with a very high AUC of 0.971, precision of 0.729, sensitivity of 0.440, specificity of 0.99, F1-score of 0.548, NPV of 0.997 and AUPRC of 0.586. The 5% births with the highest predicted risk of neonatal death included more than 90% of the actual neonatal deaths, which may help to identify focused priorities for interventions. On the other hand, there were no deaths among the 5% births with the lowest predicted risk.

**Table 1:**
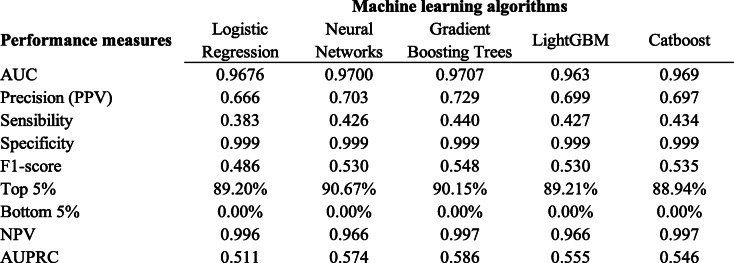
Predictive performance for neonatal mortality on the test set for each machine learning algorithm, São Paulo, Brazil, 2017

**Graph 1:**
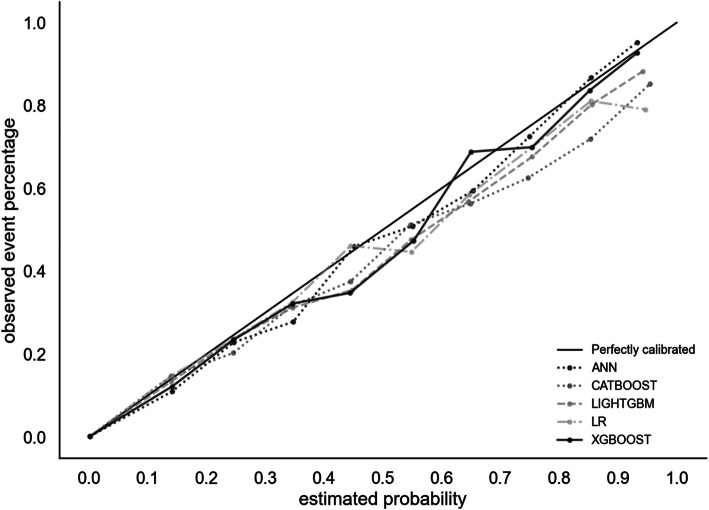
Calibration plot for neonatal mortality on the test set for each machine learning algorithm, São Paulo, Brazil, 2017

Graph 1 presents the calibration curve results for each of the machine learning algorithms. Overall, all the five models presented high calibration, meaning that the predicted risk matches the real percentage of cases. For example, for the extreme gradient boosting trees algorithm, there was a 93% mortality rate for newborns with a 90 to 95% predicted mortality risk, and an 8% mortality rate for newborns with a 5 to 10% predicted mortality risk. We also analyzed feature importance using the Shapley Values for the best-performing algorithm (extreme gradient boosting trees) and found that the five most important variables were 5th minute Apgar, birth weight, 1st minute Apgar, presence of congenital anomaly and gestational age, respectively (Additional file 1).

Table 2 presents the performance of the extreme gradient boosting trees algorithm separately for each vulnerable subgroup. The results indicate that there were no significant differences in the AUC between each complementary group: white mothers vs. non-white, adolescent mothers vs. non-adolescent, and mothers with low education vs. with average/high education. Regarding other metrics, for non-white mothers, the results of precision, sensitivity and F1-score were better than for white mothers, while for adolescents it was the opposite. Finally, the results for low education were mixed.

**Table 2:**
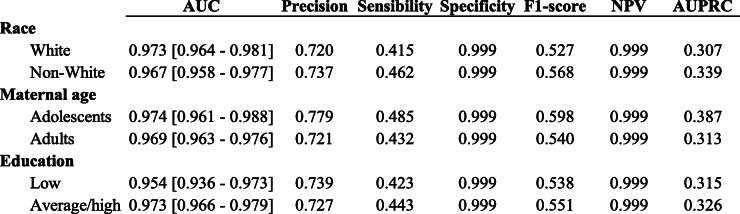
Predictive performance on the test set for selected vulnerable subgroups, São Paulo, Brazil, 2017

**Graph 2:**
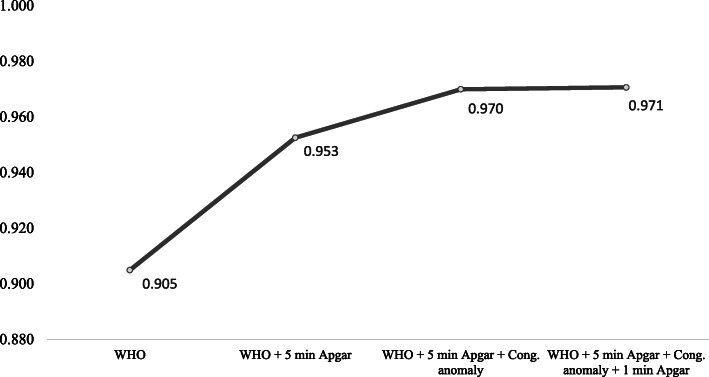
Results for the areas under the ROC Curve for neonatal mortality on the test set with the addition of variables, São Paulo, Brazil, 2017

Graph 2 shows the results of the AUC and F1-score when we included as predictors only WHO’s minimum set of perinatal indicators (AUC = 0.905 and F1-Score of 0.432). This result improves significantly with the inclusion of the 5-min Apgar score (AUC = 0.953, F-score = 0.489), with a proportionally smaller increase for the addition of congenital anomaly information (AUC = 0.970, F-score = 0.529) and first-minute Apgar score (AUC = 0.971, F-score = 0.534). Full results of the performance metrics for each addition can be found in Additional file 1.

We also performed the same analyses for infant mortality (< 1 year-old mortality). Although the AUC and F-score results were better for neonatal mortality than for infant mortality (AUC = 0.971 and F1-score of 0.548 for neonatal and AUC = 0.942 and F1-score = 0.477 for infant), both predictive performances can be considered high. Regarding the use of only the minimum set, there was also an initial decrease in the indicators, which was again reversed with the addition of three variables (Additional file 1).

## Discussion

The results show that it is possible to predict with very high performance the risk of neonatal mortality using data routinely collected in the largest city of the Americas. The use of a smaller number of variables (the five minimum perinatal indicators) decreased the predictive performance (a decrease of 6.8% of the AUC, from 0.971 to 0.905), but the results still remained high. With the addition of only three more variables (1st and 5th minute Apgar, and presence of congenital anomaly), it was possible to achieve the same predictive performance as using the 23 variables available from the Brazilian birth records.

There is a growing concern that recent machine learning breakthroughs are not executable at the frontlines of clinical practice, as most healthcare organizations do not have the infrastructure to collect the variables needed to train the algorithms [13]. Our study tackles this limitation by using only routine data collected by the health system. Despite the very high socioeconomic inequality in the city of São Paulo [14], 99.8% of all births have a birth record, and its reliability rate is considered to be very high [8]. In addition, the fact that we used data from the last year of the study (2017–2018) to test the performance of the algorithms, instead of using data drawn from the same period for training and testing, as is often the case in machine learning studies, helps to simulate its real predictive performance.

Another area of growing interest in the machine learning literature is testing the fairness of the algorithms, especially regarding classification parity, i.e. ensuring that predictive performance measures are similar across groups with vulnerable attributes [15]. Previous studies show that machine learning algorithms can be biased towards privileged groups especially due to the higher quality of data collection and the availability of more examples to guide the learning process [16]. Our analysis found that despite a slightly better result for some of the privileged groups, the specific performance for vulnerable groups were well within the margin of error.

An important future challenge for the practical application of machine learning in routinely collected data will be to define whether risk scores will be provided for all cases, or only for the highest risk patients in order to mitigate alert fatigue [17, 18]. Our study provides promising results for both possibilities. Due to the imbalanced nature of the dataset, low calibration could have been an issue but we found that the predicted risk was close to the real percentage of cases throughout the entire distribution. We also tried to mitigate this issue by analyzing the 5% births with the highest predicted risk of neonatal death and found that it included more than 90% of the actual neonatal deaths.

It is not clear that algorithms trained for São Paulo will have the same predictive performance for other cities, but we have no particular reason to think that this is a characteristic of only São Paulo. An important scientific challenge for the next years in machine learning for healthcare will be to test whether the same algorithm developed for one city or country would have similar performance in other areas, or if it is necessary to develop a new algorithm even in the case where there is less available data for training [19].

The availability of enough predictive variables is another challenge for the application of these algorithms, especially in developing countries [20]. However, our analyses show that despite the initial decrease of predictive performance when using only WHO’s five perinatal indicators, the addition of just three variables increases the performance to the same level as using the 23 variables originally available from the Brazilian birth records.

Humans face their lifetime highest risk of dying in the first month of life, with a global neonatal rate of 18 deaths per 1000 live births [3]. Identifying newborns with a high mortality risk can be the first step towards adopting targeted interventions to prevent its occurrence. Our study shows that popular machine learning algorithms are able to identify the neonatal mortality risk of newborns with a very high predictive performance using only routinely collected data.

## Supplementary Information

**Additional file 1.**

## Data Availability

Data is available in: https://bit.ly/36K8X9n.
