# Supplementary material for: Neonatal mortality prediction with routinely collected data: a machine learning approach
Source: BMC Pediatr. 2021 Jul 21;21:322. doi: 10.1186/s12887-021-02788-9 (PMC8293479; doi:10.1186/s12887-021-02788-9)

**Appendix Table 1** – Hyperparameters tested with 10-fold cross validation and final decision, São Paulo, Brazil, 2017.

| **Model** | **Hyperparameter** | **Definition** | **Range Explored** | **Best value (All features)** |
| --- | --- | --- | --- | --- |
| XGBoost | max_depth | Maximum tree depth for base learners | [3,4,5,6,7] | 5 |
|  | learning_rate | Boosting learning rate | [0.005, 0.015, 0.025, 0.035, 0.045, 0.055, 0.065, 0.075, 0.085, 0.095, 0.105, 0.115, 0.125, 0.135, 0.145, 0.155, 0.165, 0.175, 0.185, 0.195, 0.205, 0.215, 0.225, 0.235, 0.245, 0.255, 0.265, 0.275, 0.285, 0.295] | 0.085 |
|  | subsample | Subsample ratio of the training instance. | [0.1 , 0.15, 0.2 , 0.25, 0.3 , 0.35, 0.4 , 0.45, 0.5 , 0.55, 0.6 , 0.65, 0.7 , 0.75, 0.8 , 0.85, 0.9 , 0.95] | 0.85 |
|  | colsample_bytree | Subsample ratio of columns when constructing each tree | [0.1 , 0.15, 0.2 , 0.25, 0.3 , 0.35, 0.4 , 0.45, 0.5 , 0.55, 0.6 , 0.65, 0.7 , 0.75, 0.8 , 0.85, 0.9 , 0.95] | 0.45 |
|  | n_estimators | Number of gradient boosted trees | [ 50, 75, 100, 125, 150, 175, 200, 225, 250, 275] | 225 |
|  | scale_pos_weight | Balancing of positive and negative weights. | [1, 2, 3, 4] | 1 |
|  | min_child_weight | Minimum sum of instance weight needed in a child | [1, 2, 3, 4, 5, 6] | 4 |
|  | gamma | Minimum loss reduction required to make a further partition on a leaf node of the tree | [0.5, 0.55, 0.60, 0.65, 0.70, 0.75, 0.80, 0.85, 0.90, 0.95, 1.0] | 0.55 |
|  | reg_alpha | L1 regularization term on weights | [0.5, 0.75, 1.0, 1.25, 1.50] | 1.25 |
| Neural Network | activation | Activation function for the hidden layer. | ['relu', 'tanh'] | tanh |
|  | solver | The solver for weight optimization. | ['adam', 'lbfgs', 'sgd'] | lbfgs |
|  | max_inter | Maximum number of iterations. | [100, 200, 300, 400, 500, 600, 700, 800, 900] | 600 |
|  | hidden_layer_sizes | Number of neurons in the hidden layer. | [5, 6, 7, 8, 9, 10, 11] | 8 |
|  | batch_size | Size of minibatches for stochastic optimizers. | [5, 10, 15, 20, 25] | 20 |
|  | learning_rate_init | The initial learning rate used. It controls the step-size in updating the weights | [0.01, 0.03, 0.1] | 0.03 |
| LightGBM | colsample_bytree | Subsample ratio of columns when constructing each tree | [0.1 , 0.15, 0.2 , 0.25, 0.3 , 0.35, 0.4 , 0.45, 0.5 , 0.55, 0.6 , 0.65, 0.7 , 0.75, 0.8 , 0.85, 0.9 , 0.95] | 0.60 |
|  | num_leaves | The maximum number of leaves of the three | [30, 31, 32, 33, …, 148, 149, 150] | 31 |
|  | reg_alpha | L1 regularization term on weights | [0.1, 0.25, 0.5, 0.75, 1.0, 1.25, 1.50] | 1.0 |
|  | reg_lambda | L2 regularization term on weights | [0.1, 0.25, 0.5, 0.75, 1.0, 1.25, 1.50] | 0.75 |
|  | scale_pos_weight | Balancing of positive and negative weights. | [1, 2, 3, 4] | 1 |
| CatBoost | Learning_rate | Learning rate | [0.015, 0.025, 0.05, 0.1, 0.2, 0.3] | 0.025 |
|  | Max_depth | Maximum depth of the tree | [3, 6, 9, 12, 15] | 6 |
|  | Leaf_estimation_interations | Number of gradient steps to compute values of the leave | [1, 5, 10] | 10 |
|  | L2_leaf_reg | L2 regularization term on weights | [1, 3, 6, 9] | 3 |

**Appendix Table 2 -** Predictive performance for neonatal mortality on the test set with the addition of new variables, São Paulo, Brazil, 2017.

| **Model** | **ROC AUC** | **Precision** | **Recall** | **Specificity** | **F1-score** | **top 5%** | **NPV** | **AUPRC** |
| --- | --- | --- | --- | --- | --- | --- | --- | --- |
| XGBoost WHO | 0.904 | 0.750 | 0.303 | 0.999 | 0.432 | 0.727 | 0.995 | 0.440 |
| XGBoost WHO + APGAR5 | 0.952 | 0.758 | 0.361 | 0.999 | 0.489 | 0.837 | 0.996 | 0.514 |
| XGBoost WHO + APGAR 5 + COGN. ANOMALY | 0.969 | 0.718 | 0.418 | 0.999 | 0.529 | 0.893 | 0.996 | 0.567 |
| XGBoost WHO + APGAR 5 + COGN. ANOMALY + APGAR 1 | 0.970 | 0.714 | 0.426 | 0.998 | 0.534 | 0.903 | 0.996 | 0.569 |

**Appendix Table 3 -** Predictive performance for infant mortality on the test set with the addition of new variables, São Paulo, Brazil, 2017.

| **Model** | **ROC AUC** | **Precision** | **Recall** | **Specificity** | **F1-score** | **top 5%** | **NPV** | **AUPRC** |
| --- | --- | --- | --- | --- | --- | --- | --- | --- |
| XGBoost WHO | 0.871 | 0.724 | 0.232 | 0.999 | 0.351 | 0.623 | 0.993 | 0.371 |
| XGBoost WHO + APGAR5 | 0.907 | 0.725 | 0.269 | 0.999 | 0.392 | 0.704 | 0.993 | 0.431 |
| XGBoost WHO + APGAR 5 + COGN. ANOMALY | 0.937 | 0.696 | 0.338 | 0.998 | 0.455 | 0.799 | 0.994 | 0.495 |
| XGBoost WHO + APGAR 5 + COGN. ANOMALY + APGAR 1 | 0.938 | 0.702 | 0.346 | 0.998 | 0.463 | 0.801 | 0.994 | 0.500 |
| XGBoost All Variables | 0.942 | 0.704 | 0.360 | 0.999 | 0.477 | 0.806 | 0.994 | 0.513 |

**Appendix Graph 1**: Variable importance according to the Shapley Values, São Paulo, Brazil, 2017,


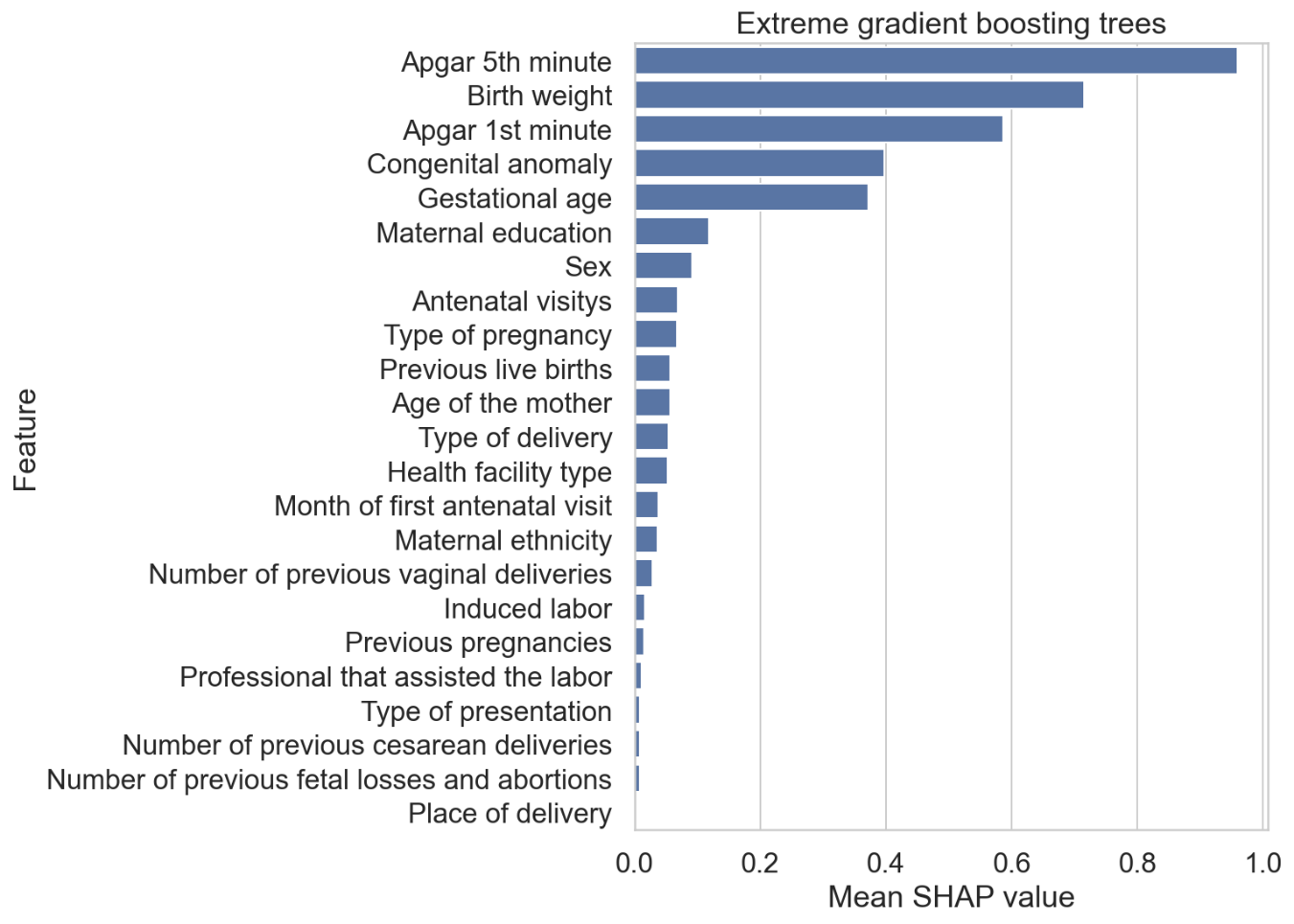

Supplement: Supplementary file 1 — Additional file 1. [file 12887_2021_2788_MOESM1_ESM.docx]
